# A Training for Hospital Executives

**Published:** 1921-04-30

**Authors:** 


### A TRAINING FOR HOSPITAL EXECUTIVES.

In the American journal, *The Modern Hospital*, for March, appeared an interesting article on "Hospital Administration in 1920," by Dr. S. S. Goldwater, the director of Mount Sinai Hospital, New York. The writer regards the chief event of the year to have been the organisation of a committee by the Rockefeller Foundation to report on "the need and practicability of a course of training for hospital executives." He believes that hospitals, in this department, show the same shortcomings as were evident in medical education before it was reformed and centralised. Unfit medical schools were then eliminated, and, it is suggested, all hospitals now must be brought, in this respect, to a fixed standard. Dr. Goldwater then turns to hospital finance, and remarks that the recent "whirlwind campaigns" of flaming publicity have lost their effect, and are leading hospitals to appoint "a permanent financial secretary at a salary approximating to that of the average high-grade hospital superintendent," and to raise patients' payments. He suggests that either scheme implies that hospital trustees are less and less disposed to raise money by their personal efforts: "The typical trustee of the day is the successful business man, whose attitude toward a hospital resembles that of the corporation director toward a business enterprise." This is said to be due to the growing complexity of hospital administration and the consequent withdrawal of the hospital trustee from its minute affairs, and "there is much to be said in favour of the shifting of responsibility for actual administration to trained, or at any rate experienced, executives. But the technique of the art of raising income perhaps better possessed by the financial secretary cannot enable him to make "the strong personal appeal" of the trustee, who can enforce it upon his friends, his business associates, and all citizens of public spirit. But the writer thinks, nevertheless, that the day of the financial secretary has come. In regard to patients for or by whom payments are made, he notes that "the principle is now recognised that in compensation cases the hospital is justified in charging a rate which will cover, in addition to current expense, the item of interest on plant cost; . . . the basis of calculation should be precisely the same as in the case of private patients. Anything less than this is equivalent to a free gift by the hospitals to the insurance companies." The plan for single rooms instead of wards is said to be too expensive to be practical, even though single rooms could be closed when not in use. We are told that one city has decided to erect a municipal hospital, because the authorities think that the private hospitals to which hitherto city cases have been sent have retained them unnecessarily long for financial reasons. The real argument for municipal hospitals, Dr. Goldwater declares, is that they will receive all classes of needy patients, whereas private hospitals prefer to limit their cases to the acutely sick and to lying-in women. It is the residuum which the private hospitals cannot conveniently admit, that the municipal hospitals can serve. He laments the lack of post-graduate teaching, and urges hospitals to provide it, and predicts that "women will play a large part in the future activities of hospitals."

In conclusion, Dr. Goldwater notes the demand for an improved method in the choice of candidates for hospital posts, and this demand is, no doubt, a contributory cause of the committee on hospital training now formed under the Rockefeller Foundation.

